# Green Synthesis of Silver Nanoparticles Using *Cynoglossum creticum* Leaf Extract: Eco-Friendly Approach for Antibacterial, Antioxidant, and Sensing Applications

**DOI:** 10.1007/s12010-025-05390-2

**Published:** 2025-10-23

**Authors:** Seif El Islam Boudagha, Chafia Sobhi, Hamdi Bendif, Emel Öykü Çetin Uyanikgil, Amdjed Abdennouri, Mustafa Ökeer, Chawki Bensouici, Moussa Boudiaf, Ahmed Zouaoui, Hassan A. Rudayni, Fehmi Boufahja, Stefanıa Garzoli

**Affiliations:** 1 Laboratoire de Physico-Chimie Des Surfaces Et Des Interfaces, Université 20 Août 1955 de Skikda, BP 26, Route El Hadaik, Skikda, 21000 Algeria; 2 Laboratory of Materials and Energetic Engineering, Faculty of Technology, University of 20 August 1955, BP 26, Skikda, 21000 Algeria; 3 Department of Biology, College of Science, Imam Mohammad Ibn Saud Islamic University (IMSIU), Riyadh, 11623 Saudi Arabia; 4 Department of Biopharmaceutics and Pharmacokinetics, Faculty of Pharmacy, Ege University, Izmir, Turkey; 5 Laboratory of Catalysis, Bioprocess and Environment, Department of Process Engineering, Faculty of Technology, University of 20August 1955, Skikda, 21000 Algeria; 6 Department of Pharmaceutical Microbiology, Faculty of Pharmacy, Ege University, Izmir, Turkey; 7 Laboratory of Biochemistry, Biotechnology and Health Division, Center for Research in Biotechnology, Constantine, 25000 Algeria; 8 LCIMN Laboratory, Department of Process Engineering, Faculty of Technology, University Ferhat Abbas Setif-1, Sétif, 19000 Algeria; 9 Laboratoire Croissance Et Caractérisation de Nouveaux Semiconducteurs (LCCNS), Université Ferhat Abbas Sétif-1, Sétif, Algeria; 10 Department of Chemistry and Technologies of Drug, Sapienza University, Rome, 00185 Italy

**Keywords:** Silver nanoparticles, *Cynoglossum creticum*, Antioxidant, Antibacterial, Colorimetric sensing

## Abstract

Nanotechnology leveraging renewable plant biomass to develop silver nanoparticles offers eco-friendly solutions with more sustainable applications. The present research explores the employment of an aqueous extract of *Cynoglossum creticum* leaves in the green synthesis of silver nanoparticles (Ccl-AgNPs) for potential biological and sensing purposes. The synthesis parameters were controlled spectrophotometrically, and the physicochemical characterizations of Ccl-AgNPs were evaluated. Phytochemicals in the extract served dual roles, as confirmed by infrared spectroscopy analysis. Thermogravimetric analysis (TGA) and energy-dispersive X-ray spectroscopy (EDS) further revealed the presence of a plant-derived organic matter on AgNPs. Morphological descriptions indicated the spherical form of Ccl-AgNPs, comprising a median size of 32 nm. Furthermore, the biological characteristics of green Ccl-AgNPs were investigated, as they showed promising antioxidant activities in DPPH, ABTS, reducing power, and phenanthroline assays. Ccl-AgNPs also exhibited a good inhibitory impact on the growth of four tested Gram-positive and Gram-negative bacteria. *Pseudomonas aeruginosa* was most susceptible to the inhibitory activity of Ccl-AgNPs, with a minimum inhibitory dose of 31.25 µg/mL. Colloidal AgNPs capped by Ccl-extract have established the potential for use as an SPR-based colorimetric sensing system for the selective detection of neomycin sulfate, with a limit of detection (LOD) of 2.88 µM. The colorimetric probe was successfully tested for neomycin sulfate detection in environmental and biological fluids and veterinary pharmaceutical preparations, with recovery rates ranging between 92.62 and 105.76%. This study underscores the potential of *Cynoglossum creticum*, a livestock-hazardous weed, as a sustainable resource for synthesizing silver nanoparticles for potential ecological and biomedical uses.

**Supplementary Information:**

The online version contains supplementary material available at 10.1007/s12010-025-05390-2.

## Introduction

The revolution in nanotechnology over the past decade has brought about a dynamic leap in human life[1]. It is a modern, multidisciplinary science, encompassing the design and manipulation of nanoscale materials with unique properties to address contemporary challenges[2, 3]. Among the nanostructures, silver nanoparticles (AgNPs) stand out as a popular choice that has earned increasing attentiveness in recent investigations due to their distinctive physicochemical and biological properties like strong light absorption, high surface energy, conductivity, chemical stability, and biocompatibility[4, 5]. These intrinsic features support their broad application in electronics, water treatment, catalysis, optics, and energy harvesting, as well as biomedical and pharmaceutical applications such as wound healing, gene and drug delivery, and biosensing[6, 7]. The rising demand for AgNPs has motivated large-scale production using various chemical and physical strategies, estimated at 550 metric tons per year[8, 9]. However, these techniques pose challenges, like ecotoxicity, cytotoxicity, genotoxicity, and carcinogenicity, as well as high costs in terms of specialized equipment, energy, and time[8, 10, 11].

Recently, several studies were dedicated to developing simple, economical, effective, low-toxicity, more eco-friendly, and reliable methods for creating silver nanostructures[12]. The biological synthesis using natural entities such as plants is a green alternative option[6, 13]. Plant and their extracts provide phytochemicals that not only reduce silver ions but also coat the resulting Ag⁰, enabling the production of stable AgNPs with varied sizes and shapes[14, 15]. Furthermore, the phytochemicals are highly effective natural antioxidants and antibacterial agents. They are rich sources of electrons that work to scavenge free radicals, prevent lipid peroxidation, quench singlet oxygen, and chelate metals, which helps reduce oxidative stress in the body[16, 17]. Interestingly, the extracts contain both lipid-soluble compounds, such as flavonoids and quinones, and aqueous-soluble compounds, such as nicotinamide, which enhance their role in protecting biological entities from oxidation[18]. Indeed, silver metals have been used for centuries to treat bacteria-related disorders[19]. Silver, in its nanoform, has proven to be an effective antibacterial agent against a variety of human and animal pathogens[11]. Notably, it shows significant potential in combating antibiotic-resistant bacterial biofilms by disrupting their structural integrity. This is achieved through interactions with eDNA, binding and preventing the fibrilization of extracellular proteins[20]. Green-synthesized silver nanoparticles offer superior biocompatibility and enhanced antioxidant and antimicrobial properties, driven by synergistic interactions between silver and the bioactive molecules used in the synthesis process[21–23]. However, despite these advantages offered by plant-based synthesis, a technical drawback is the difficulty in determining the synthesis and stabilization mechanisms and precisely controlling the morphology of nanoparticles. This is primarily due to the vast diversity of phytochemicals present in the extracts and involved in the process[24]. Optimizing and improving synthesis parameters significantly helps overcome this drawback.

Neomycin sulfate is one of the most widely applied aminoglycoside antibiotics in veterinary medicine for the treatment of bacterial infections in animals and livestock, such as scours (bacterial enteritis), mastitis, and hepatic encephalopathy[25, 26]. It is also incorporated with feed and milk replacer to enhance prophylaxis and growth[25]. However, misuse of neomycin sulfate, as well as its excessive and illegal addition to feed, can result in its transfer to waterbodies, nearby land, and animal products as drug residues[27, 28]. Like all antibiotics, environmental exposure promotes the growth of antibiotic-resistant bacteria and induces genome changes responsible for mutations, increasing their risk[29]. In terms of human health, neomycin sulfate may penetrate and accumulate in the human body system via the nutrition cycle and induce significant adverse consequences like anaphylaxis, tissue toxicity, and neurotoxicity[30, 31]. Long-term accumulation of neomycin causes deafness, cardiovascular disease, and kidney damage[31, 32]. It is of practical importance to monitor and mitigate the potential effects of neomycin sulfate by developing sensitive analytical procedures for its qualitative and quantitative detection. Nanotechnology offers a wealth of highly sensitive sensing methods that can accurately identify an analyte in a complex environment[33]. Colorimetric sensing, which leverages the surface plasmon resonance (SPR) properties of noble metal nanoparticles, especially silver nanoparticles, is one of the most prominent approaches[34]. The importance of silver nanosensors lies in their superior optical properties and extinction coefficients, allowing them to detect a wide range of analytes, including heavy metals, amino acids, and pharmaceutical compounds[31, 35].

*Cynoglossum creticum*, frequently called blue hound’s tongue, is an erect, branched biennial plant that is affiliated with the *Cynoglossum* L. genus in the Boraginaceae family, and is widely spread throughout the Mediterranean basin[36]. This wild plant thrives in dry and open habitats on the edges of forests, fields, and roadsides. *Cynoglossum creticum*, like the majority of the *Cynoglossum* L. genus, is abundant in hepatotoxic pyrrolizidine alkaloids (PAs), which have been classified as a harmful weed to horses and livestock in many countries[36, 37]. Nonetheless, *Cynoglossum creticum* was employed in the traditional medicine of the Mediterranean peoples as a wound poultice and in treating cold head and throat infections such as purulent boils[16]. The phytochemical profile of *Cynoglossum creticum* has been reported, and its multiple pharmacological potential has also been investigated[34, 35]. *Cynoglossum creticum* leaves are high in natural bioactive substances, including phenolic acids and allantoin, which offer them significant antioxidant and enzyme inhibitor properties[16, 38]. Numerous research studies have highlighted the use of extracts from various members of the Boraginaceae family in the biosynthesis of AgNPs, which exhibit unique structural, biological composition, and therapeutic properties[39, 40]. For instance, J. Kamalanathan et al.[41] revealed the biomedical potential of AgNPs produced from oil-rich aqueous extracts of *Cynoglossum furcatum* leaves, as they showed biocompatibility and high efficacy against cancer cell lines while exhibiting low cytotoxicity to normal cells. Similarly, *Anchusa azurea* flower extracts have been employed in the fabrication of silver nanoparticles for the colorimetric sensing of Hg^2^⁺ ions in aqueous media[42]. However, despite substantial research into the significance of the Boraginaceae family in the biosynthesis of nanomaterials, the application of *Cynoglossum creticum* in bio-nanotechnology has not yet been explored.

This research aims to valorize *Cynoglossum creticum* leaves by highlighting the possibility of using their aqueous extract in the biomanufacturing of silver nanoparticles (Ccl-AgNPs). The process was improved via examining the impact of various factors, and the optimized AgNPs were analyzed by spectroscopic and imaging approaches. Furthermore, the biopharmaceutical properties of Ccl-AgNPs as antioxidants and antibacterials were assessed, as well as their potential as an LSPR-based chemosensor for sensitive and selective detection of the antibiotic neomycin sulfate in real ecological, biological, and veterinary drug samples.

## Experimental

### Chemicals and Drugs

In order to properly carry out our investigation, chemicals and reagents of analytical research grade were used. The items listed below were provided by Sigma-Aldrich and Fluka Chemie: silver nitrate (BP USP 99.8–100.5%), 2,2-diphenyl-1-picrylhydrazyl (DPPH), *α*-tocopherol, butylated hydroxyanisole (BHA), ascorbic acid, butylated hydroxytoluene (BHT), 2,2′-azino-bis(3-ethylbenzothiazoline-6-sulfonic acid) (ABTS), potassium ferricyanide, potassium persulfate, trichloroacetic acid (TCA), ferric chloride, 1,10-phenanthroline, catechin, Folin–Ciocalteu reagent, sodium carbonate, gallic acid (GA), aluminum nitrate, potassium acetate, and quercetin. As for the drugs used in the investigation, including salbutamol sulfate, propantheline bromide, neomycin sulfate, teofilin, sodium valproate, atenolol, dextromethorphan hydrobromide, Nipagin M, diclofenac sodium, caffeine, paracetamol, and capecitabine, they were provided by the İlsan İlaç ve Hammaddeleri Sanayi Ticaret A.Ş, Türkiye, and BOC Sciences.

### Cynoglossum creticum Extract Preparation

*Cynoglossum creticum* basal leaves were picked in April from the Azzaba region wilaya of Skikda, northeastern Algeria. Then they were properly cleansed using faucet water and then distilled water to eliminate dust and dirt. The leaves were then spread and dried in the shade under ventilation conditions to prevent the loss of bioactive compounds. After checking that the leaves were drying, they were well-ground into a homogeneous powder using an electronic grinder and stored for subsequent use in an airtight container in a dry dark location. Two grams of the resulting powder was mixed with 200 mL of distilled water (i.e., 1% w/v) in a 250-mL conical flask under continuous stirring and boiling for 1 h at 70 °C. The mixture was then cooled to room temperature before being filtered with filter paper no. 1 and maintained at 4 °C.

### Phytochemical Analysis

To figure out the bioactive components that cause bio-reduction and coating, preliminary plant-specific tests were performed on the powder or aqueous extract of *Cynoglossum creticum* leaves using standard qualitative methods based on monitoring color changes and the development of precipitations. Screening tests were performed for tannins[43], alkaloids[44], saponins[43], flavonoids[43],quinones[45] anthraquinone[43], and sterols and terpenes[46]. For reliability, the experiments were repeated.

### Biofabrication of Silver Nanoparticles (Ccl-AgNPs)

Ccl-AgNPs were produced in a one-step green way using *Cynoglossum creticum* leaves extract as a dual-role agent. Briefly, the Ccl-extract was introduced to a 250 mL-conical flask including 1 mM of aqueous silver nitrate solution and continuously stirred till the mixture’s color altered from pale yellow to dark brown. To identify ideal circumstances for synthesis, the impact of variables like the volume ratio of AgNO_3_/Ccl-extract, temperature (room temperature to 80 °C), and reaction time was monitored, employing a UV–vis spectrophotometer (UV-1900i, Shimadzu Corporation), by measuring the absorption spectra of the silver precursor-extract reaction mixture.

### Ccl-AgNPs Characterization

The green-synthesized Ccl-AgNPs were assessed using characterization techniques listed below. Fourier transform infrared (FTIR) spectra were recorded in the wavelength range from 4000 to 400 cm^−1^ employing a QATRTM-S single-reflection ATR accessory with a diamond crystal (Shimadzu Corporation, Japan). X-ray diffraction (XRD) investigations were assisted by a Bruker D8 ADVANCE with DAVINCI design (Bruker AXS, Germany) operating at a current of 40 mA and a voltage of 40 kV, with a Cu K*α* radiation of wavelength 1.54 Å in a 2*θ* range of 10–90°, a step size of 0.02°, and a total time/step of 19.1 s. A morphological and elemental composition study was performed utilizing environmental scanning electron microscopy (ESEM) packed with energy dispersive X-ray spectroscopy (EDS) measurements (Quattro S-ESEM, Thermo Fisher Scientific, Waltham, MA, USA). Heat resistance was assessed through thermogravimetric analysis using a Mettler Toledo TGA–DSC 3 + Thermal Analyzer (Mettler Toledo, Switzerland) under a nitrogen atmosphere, at a heating rate of 10 °C/min from ambient temperature to 1000 °C.

### Screening of Antioxidant Properties

#### Determination of Total Phenolic and Flavonoid Contents

Total polyphenol content (TPC) of Ccl-AgNPs was estimated utilizing the standardized Folin–Ciocalteu approach[47]. Briefly, 20 µL of 1 mg/mL of aqueous colloidal solution of Ccl-AgNPs was introduced to 100 µL of Folin–Ciocalteu reagent (diluted tenfold in distilled water) and 75 µL of 75 g/L sodium carbonate solution. The mixture was kept to incubate in the dark for 2 h at room temperature prior to assessing the absorbance at 765 nm in a 96-well microplate reader (En Spire, PerkinElmer, MA, USA). TPC was identified via the gallic acid standard and displayed as micrograms of gallic acid equivalents per milligram of Ccl-AgNPs (µg GAE/mg dried Ccl-AgNPs).

Total flavonoid content was determined through the AlCl_3_-flavonoids complexation colorimetric method[48]. In total, 130 µL of methanol was introduced to the microplate (96 wells), comprising 50 µL of (1 mg/mL) Ccl-AgNPs, followed by the injection of 10 µL of 1M potassium acetate and 10 µL of 10% aluminum nitrate. The plate was incubated for 40 min at ambient temperature. Absorbance was assessed at 415 nm, and TCF was stated as micrograms of quercetin equivalent/milligram of dry weight nanoparticles (µg QE/mg Ccl-AgNPs).

#### DPPH Radical Scavenging Activity Test

Ccl-AgNPs’ activity in scavenging DPPH free radicals was evaluated spectrophotometrically by the method described by M. Blois[49]. In a 96-well microplate, 0.1 mM of methanolic DPPH solution and different concentrations of redispersed Ccl-AgNPs in distilled water were mixed with a volume ratio of 4:1 (DPPH/Ccl-AgNPs), preserved in darkness at ambient temperature for 30 min; the absorbance was measured at 517 nm. Antioxidant standards used for comparison included *α*-tocopherol, BHA, and BHT. Outcomes were represented as 50% inhibitory concentration (IC_50_), which is the Ccl-AgNPs’ concentration (µg/mL) necessary to scavenge DPPH free radicals by 50%.

#### ABTS Cation Radical Assay

The antiradical activity of the Ccl-AgNPs in decolorizing the cationic radical ABTS was investigated according to Re et al.’s[50] process, with slight alterations. First of all, ABTS^•+^ solution was arranged by mixing 7 mM of an aqueous solution of ABTS with 2.45 mM of potassium persulfate. The mixture was then left in darkness for 16 h before being diluted to get an absorbance of 0.700 at 734 nm. Next, 40 µL of different doses of Ccl-AgNPs were mixed with 160 µL of the previously prepared solution and incubated in darkness for 10 min, and the absorbance was detected at 734 nm. Findings were given as inhibition at 50% concentration (IC_50_), with BHT and BHA utilized as antioxidant standards to compare the activity of the Ccl-AgNPs.

#### Reducing Power Assay

Ccl-AgNPs reducing power was evaluated based on the previously described Oyaizu[51] protocol, with minor changes. A reaction mixture including 10 µL of different Ccl-AgNPs doses, 40 µL of 200 mM phosphate buffer (pH 6.6), and 50 µL of 10 g/L potassium ferricyanide was incubated for 20 min at 50 °C. Subsequently, 50 µL of trichloroacetic acid solution (10%) was added to the mixture, followed by 40 µL of distilled water and 10 µL of ferric chloride solution (1.0 g/L). Ultimately, the absorbance was highlighted at 700 nm; utilizing linear regression analysis from the calibration curve, the sample concentration corresponding to the absorbance intensity of 0.50 (A_0.5_; µg/mL) was calculated. Antioxidant standards were also used as follows: ascorbic acid, *α*-tocopherol, catechin, and BHT.

#### Phenanthroline Test

The reducing activity was also examined by the 1,10-phenanthroline approach described by Szydłowska-Czerniak et al.[52], with slight changes. Catechin, BHA, ascorbic acid, and BHT were chosen as positive controls. In summary, 50 µL of ferric chloride (0.2%) and 30 µL of *O*-phenanthroline (0.5%) were introduced to 10 µL of samples at various concentrations, then methanol was added until the mixture volume reached 200 µL. The reaction mixture was allowed to incubate in the dark at 30 °C for 20 min before measuring the absorbance at 510 nm. Findings are highlighted as *A*_0.50_ (µg/mL), which represents the concentration at which any absorption of 0.50 occurs.

### Antibacterial Activity of Ccl-AgNPs

Antimicrobial activity of Ccl-AgNPs was assessed by assuming the minimum inhibition concentration (MIC) via the broth microdilution technique. The test was performed against four bacterial strains supplied by the Laboratory of Pharmaceutical Microbiology at Ege University, namely *Enterococcus faecalis* ATCC 29212, *Staphylococcus aureus* ATCC 29213, *Escherichia coli* ATCC 25922, and *Pseudomonas aeruginosa* ATCC 27853. Before screening, microorganism strains were reactivated in a Mueller–Hinton broth (MHB) medium at 37 °C for 18 h, the time required for growth to reach the exponential phase. The cell density of culture suspensions was then adapted to 0.5 McFarland standard utilizing physiological saline solution, which was subsequently diluted in a ratio of 1/100 to achieve a final concentration of 5 × 10[5] CFU/mL.

On sterile 96-well plates containing 50 µL of MHB, serial dilutions of redispersed Ccl-AgNPs were made, starting by adding 50 µL of the colloidal solution to the first well of the plate and completing twofold dilution to obtain concentrations between 15.62 and 1000 µg/mL. Overall, 50 µL of microorganism suspensions were introduced to each well, and the microplates were put in an incubator at 37 °C for 24 h. After the incubation period, the concentrations that inhibited the growth of bacteria in the wells were visually observed, and the fewest Ccl-AgNPs concentration ensuring microorganism growth inhibition was determined as MIC values.

### Colorimetric Detection of Pharmaceutical Drugs

To assess the analytical applications of Ccl-AgNPs in the chemosensing ability of neomycin sulfate (NEO) and other pharmaceutical drugs, including salbutamol sulfate, propantheline bromide, teofilin, sodium valproate, atenolol, dextromethorphan hydrobromide, Nipagin M, diclofenac sodium, caffeine, paracetamol, and capecitabine, 100 µg/mL of colloidal solution of Ccl-AgNPs and 1 mM aqueous solutions of the drugs were mixed in test tubes in equal volume ratios of 1:1 (v/v) at room temperature for a few minutes. The initial interactions were tracked visually by color change and spectrophotometrically by recording the variation in the SPR range.

The applicability of the Ccl-AgNPs-based colorimetric approach for detecting NEO was estimated in different mediums. Samples of tap water were taken from Ege University, Izmir, Turkey, and used without any additional treatments in preparing different concentrations of neomycin sulfate (10, 30, and 50 µM). Subsequently, by the same principle, NEO solutions were mixed with Ccl-AgNPs solutions at a 1:1 v/v ratio, and changes in the absorption intensity and SPR band were monitored. Likewise, blood samples from healthy rabbits were collected at the research laboratory of the Department of Biopharmaceutics and Pharmacokinetics at Ege University. The samples were centrifuged at 4000 rpm for 20 min at 25 °C to separate the blood parts and extract the plasma. Before performing the sensing test, the plasma was diluted 100 times with distilled water and spiked with different amounts of NEO to get final concentrations between 10 and 50 µM. Then, an equal volume of Ccl-AgNPs was inserted into the spiked plasma samples, and the UV–vis spectra were noted. The neomycin sensing response was also monitored in veterinary medicine products. A commercially available Neoxyvit-AL drug in the form of a powder containing 30 mg/g of neomycin sulfate was employed to get solutions with various concentrations of NEO. The solutions were then treated with colloidal Ccl-AgNPs, and the changes in the UV–vis spectrum were measured.

## Results and Discussion

### Preliminary Phytochemicals Screening of Ccl-Extract

Phytochemical screening is a basic, uncomplicated approach for detecting numerous primary and secondary metabolites and is one of the most significant steps in the study of plants, especially medicinal ones[53]. Phytochemicals are natural, biologically active compounds available in plants that, along with nutrients and fiber, form a protection system against diseases[54]. A preliminary phytochemical examination was done to ascertain the metabolites present in *Cynoglossum creticum* leaves aqueous extract, and the results (Table S1) reveal the availability of tannins, alkaloids, flavonoids, quinones, saponnins, and steroids/terpenes, as well as the absence of anthraquinon.

The results demonstrate the abundance of medically active secondary metabolites in the aqueous extract of *Cynoglossum creticum*; this indicates the usage of this plant in traditional folk medicine. Alkaloids, flavonoids, quinones, and tannins have anticholinesterase, analgesic, antibacterial, and immunomodulatory activities, as well as being effective in preventing age-related toxicological pathways associated with neurodegenerative diseases[55, 56]. Saponins play an important role as pre-formed barriers against pathogens. Water-soluble saponins can also act as inhibitors of induced defense responses[57]. Furthermore, these secondary metabolites are biocatalysts that possess reducing groups that can reduce metal ions and produce nanoparticles by donating hydrogen atoms or electrons.

### Biosynthesis and Optimization of Ccl-AgNPs

The feasibility of synthesizing silver nanoparticles using Ccl-extract without using any additional chemical reactants was examined. In the experiments, there was a rapid and gradual modification in the reaction solution color, as the pale yellow resulting from mixing both the aqueous solution of AgNO_3_ (1 mM) and Ccl-extract gradually turned to reddish brown and then blackish brown within minutes (Fig. S1). The brown color demonstrates the development of AgNPs in the colloidal, where, because of the optical properties of AgNPs, electromagnetic waves are absorbed with a specific frequency caused by oscillations of the surface electron[58]. It is recognized as the surface plasmon resonance (SPR) phenomenon, which is associated with this color appearance.

**Fig. 1:**
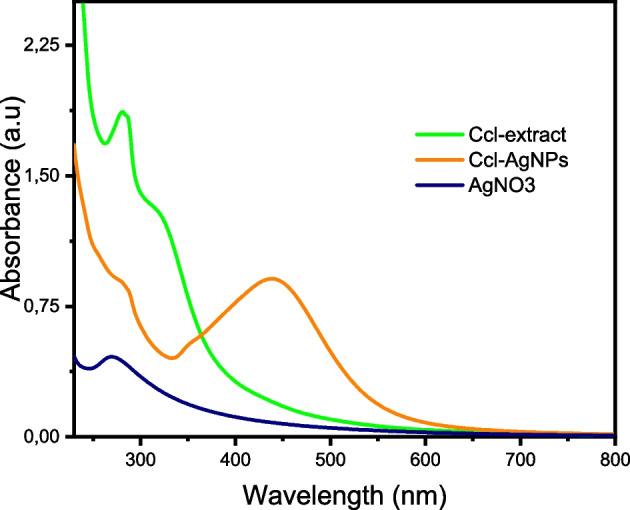
UV–vis spectra of *C. creticum* leaves extract, silver nitrate, and Ccl-AgNPs

Furthermore, UV–visible spectroscopy was employed to follow up the initial screening of silver nanoparticles during the synthesis process. Figure 1 displayed the UV–Vis spectra of the Ccl-extract, silver precursor, and Ccl-AgNPs from 200 to 800 nm. The absorption spectrum of the Ccl-extract stated an absorption peak at 281 nm and a hump at about 325 nm due to the π–π* and n–π* transitions related to the C = C, carbonyl, and hydroxyl groups of the alkaloid and phenolic components found in the leaves of *Cynoglossum creticum*[59]. The spectrum of AgNO_3_ revealed a strong absorption peak in the UV region at 269 nm. In contrast, the spectrum of the colloidal Ccl-AgNPs exhibited a prominent absorption peak at around 435 nm with a small hump at 350 nm on account of the surface plasmon resonance characteristic. The SPR peak typically appears in the range between 350 and 450 nm, and its *λ*_max_ and intensity rely on the size, form, and dissemination of AgNPs, which are affected by changes in parameters such as reactant concentrations and temperatures[58]. Therefore, the role of each reaction parameter was evaluated applying a one-variable-at-a-time technique in order to achieve the maximum yield of Ccl-AgNPs with adequate stability and dispersion.

### The Amount of Silver Nitrate Impact

**Fig. 2:**
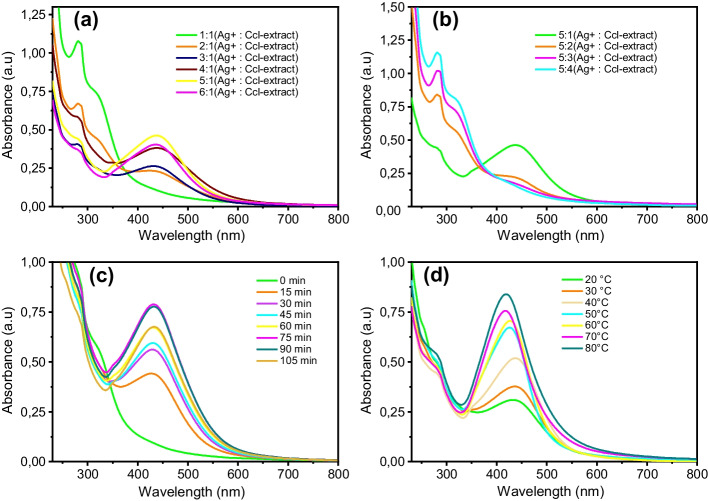
UV–vis spectra of synthesized Ccl-AgNPs under experimental parameters: **a** as a function of the amount of silver nitrate, **b** as a function of the concentrations of Ccl-extract added, **c** as a reaction time, and **d** at various temperatures

The AgNO₃ solution volume’s impact on the synthesis was studied by changing the volume ratio of AgNO_3_ in the mixture while maintaining the volume of the added extract constant (from 1:1 to 6:1; AgNO_3_/Ccl-extract) and stirring the mixture for an hour at 50 °C. Figure 2a depicts the UV–vis spectra of Ccl-AgNPs prepared with different volumes of silver precursors. The absorption spectrum of (1:1) was similar to the Ccl-extract spectrum (Fig. 1), where the absorption peak at 218 nm remained, and no peak appeared in the SPR region, suggesting that AgNPs did not form. The strength of the peak at 281 nm lowered as the level of silver ions increased, and a novel absorption peak at roughly 435 nm developed, with its intensity and sharpness increasing to reach the highest with a 5:1 ratio (AgNO_3_/Ccl-extract). However, the absorption spectrum of AgNPs prepared using volume ratios (AgNO_3_/Ccl-extract) greater than 5:1 revealed a SPR peak with lower intensity.

### Ccl-Extract Concentration Impact

Given the parallel function of Ccl-extract as a reducing and capping agent for nanoparticles, finding its appropriate concentration to ensure silver ion reduction and achieve sufficient stability of the developed AgNPs is critical. Following the same principle, a constant volume of 1 mM of silver nitrate was treated with various Ccl-extract solution ratios (5:1 to 5:4; Ag^+^/Ccl-extract) under the same previous conditions, i.e., time and temperature.

From Fig. 2b, the rise in the concentration of the Ccl-extract seen may lead to a severe reduction in the absorption intensity at 435 nm until it vanished completely at a ratio of 5:4 (Ag^+^/Ccl-extract), demonstrating that Ag^+^ ions were not reduced. Plant leaves are known to be the most important source of phenols and flavonoids, which in turn have redox properties that allow them to reduce silver ions by donating electrons and also serve a stabilizing role for the generated AgNPs by covering their surface[60]. However, the high concentration of these compounds leads to the interaction between their functional groups instead of an interaction with saturated silver ions, thus resulting in an Ag-hydro-complex rather than a colloidal solution of AgNPs[61]. Based on these findings, a ratio of 5:1 (Ag^+^/Ccl-extract) was determined to be optimal and chosen for further studies.

### Reaction Time Effect

The influence of contact time on the reduction reaction was explored to determine the yield point of reduction that ensures complete nucleation and particle stability. Figure 2c presents the reaction mixture UV–vis spectra during the synthesis at multiple times in the range of 0–105 min with 15-min intervals. It is evident that the SPR band of AgNPs appeared within 15 min of the reaction; however, the band was broad, weak, and of low intensity. The SPR band severity improved as the incubation time expanded, up to 75 min. Increases in absorbance values correlate to increases in the concentration and size of AgNPs in colloidal solution. The additional extension in reaction time culminated in a considerable decline in the absorption intensity with a slight redshift, especially when up to 105 min of reaction. This could be as a result of the agglomeration of nanoparticles and the decrease in stability of the nanosystem caused by the evolution of the crystal faces (1 1 1) by deposition of Ag atoms on the cubic faces (1 0 0) rather than the nucleation of new crystals[62]. Thus, 75 min was determined to be a suitable duration for Ccl-AgNPs production.

### Temperature Effect

The reaction temperature affects nanoparticles’ formation in terms of size and shape. The temperature-dependent Ccl-AgNPs synthesis was performed in the domain of room temperature to 80 °C under other optimal parameters. According to Fig. 2d, the SPR band peak appeared at all temperatures examined, proving the formation of Ccl-AgNPs. Temperature rise from room temperature to 80 °C was accompanied by an increase and broadening in the absorption spectrum, with a blue shift in the *λ*_max_ values from 438 to 424 nm. The broadening in the absorption spectrum can be attributed to the enhanced phonon-electron scattering rate[63]. While the wavelength lowering is related to the fact that smaller and more stable spherical AgNPs were formed at higher temperatures as more energy was provided to the reaction, which boosted the efficiency of Ag^+^ ion reduction and subsequent silver nuclei nucleation and nanoparticle formation[64]. In the biosynthesis of nanoparticles, low temperatures favor growth and increase in particle size, whereas high temperatures favor nucleation[65]. Therefore, the most appropriate temperature for Ccl-AgNPs manufacturing is considered to be 80 °C.

In this work study, the perfect setting for the formation of Ccl-AgNPs for future characterization and application was found to be 1 mM of silver nitrate against Ccl-extract with a volume ratio of 5:1 (AgNO_3_/Ccl-extract), 80 °C temperature, and 75 min incubation time. After synthesis, the solid Ccl-AgNPs were recovered by centrifuging the colloidal solution for 20 min, rinsing it three times with distilled water, and allowing the Ccl-AgNPs to dry fully at 60 °C. Additionally, the slight hump at about 350 nm disappeared from the absorption spectrum of the optimum Ccl-AgNPs (Fig. S2), and the SPR peak became single, sharper, and nearly symmetrical, indicating the formation of monodispersed spherical nanoparticles[66].

### Stability of Ccl-AgNPs

The applicability of AgNPs is related to their stability in environments that actually contain other intervening substances, such as ions. Electrolytes’ effect on the stability of the developed Ccl-AgNPs was assessed by treating Ccl-AgNPs with various doses of NaCl aqueous solution (0.0001 to 2 M) and recording UV–vis spectra. Based on Fig. S3, the intensity of the absorption SPR peak decreased with rising electrolyte concentrations. This decrease can be linked to the aggregation of nanoparticles resulting from the reduction in electrostatic repulsion and surface charge of AgNPs in the presence of free Cl^−^ ions[67, 68]. The Ccl-AgNPs showed stability even up to 1 M NaCl, as there was a sharp decrease in the absorption intensity with increasing ionic strength up to 2 M. According to the literature[69], the normal range of chloride in human blood has been reported to be 96–108 (mEq/L), which proves the applicability of biosynthesized Ccl-AgNPs for biological and environmental samples.

Salts and other interferences’ presence in real samples may lead to a change in pH, impacting the stability of the AgNPs. Varying pH impact on the SPR range was examined by adjusting the pH of Ccl-Ag-NPs solutions between 2 and 10 by dropping 1 M of HCl or NaOH solution. Synthesized Ccl-AgNPs are mildly acidic in nature (pH about 5); however, the SPR peak intensity decreased with decreasing pH (Fig. S4), and a precipitate formed in highly acidic conditions at pH 2. This indicates the destabilization and aggregation of the nanoparticles, which may result from the neutralization of the surface charge of the AgNPs due to the removal of the stabilizing phytochemicals[70]. Moreover, the increase in pH increased absorption peak intensity, i.e., enhancing the colloidal Ccl-AgNPs’ stability in the basic medium, which may be associated with OH^−^ which could operate as ligands[71].

### Biosynthesized Ccl-AgNPs Characterization

#### FTIR Spectroscopy

**Fig. 3:**
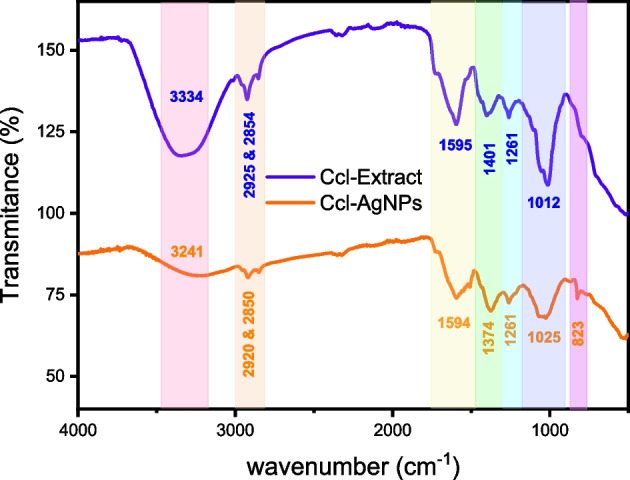
FTIR spectra of *C. creticum* leaves extract and biogenic Ccl-AgNPs

FTIR spectra aid in identifying probable functional groups of secondary metabolites found in the Ccl-extracts that took part in the Ag^+^ ions bioreduction process and the surface coating of Ccl-AgNPs. The spectrum of the leaf extract (Fig. 3) reveals the presence of phenolics, which provide antioxidant and antibacterial characteristics to the extract. The broad band at 3334 cm^−1^corresponded to O–H stretching vibrations of alcohols, phenols, carboxylic groups, and intramolecular H bonds from water molecules. Two absorption bands at 2925 and 2854 cm^−1^ may be ascribed to asymmetric and symmetric stretching vibrations of methylene groups (CH_2_ and CH_3_). A band at 1595 cm^−1^ highlights the existence of phenolic and protein compounds since it corresponds to the C = C stretching vibration of the aromatic ring. The band at 1401 cm^−1^ may be attributed to C–H deformation of alkane and alkene or C–C stretching of the aromatic ring[72]. Characteristic peaks at 1261 and 1012 cm^−1^ may be related to the C–N stretching vibrations of aromatic and aliphatic amines, respectively [73, 74]^74,75^.

FTIR spectrum of Ccl-AgNPs (Fig. 3) is remarkably akin to Ccl-extract one, with the exception of a shift in the position and intensity of the bands. This change is caused by the silver ions’ interaction with functional groups of phytocompounds; this leads to the development of nanoparticles which are stabilized from agglomeration by the remaining phytochemicals, confirming the extract’s combined action as a reducing and stabilizing agent. The broad peak of hydroxyl groups and C–H stretching peaks in the FTIR spectrum of Ccl-AgNPs shifted to lower values with low intensity of 3241, 2920, and 2850 cm^−1^, respectively, due to the lesser number of moieties associated with the produced AgNPs compared to those in the extract[75]^76^. The literature has proven the essential role of hydroxyl groups during the biosynthesis process, where silver ions Ag^+^ are reduced to Ag^o^ by transferring electrons resulting from hydrogen bond breaking and oxidation of hydroxyl groups to carbonyl groups (R–C = O)[76, 77]. Characteristic peaks at 1401 and 1012 cm^−1^ also shifted to 1374 and 1025 cm^−1^, respectively, confirming the involvement of aliphatic and aromatic groups of proteins and flavonoids in the biosynthesis process. In addition, a new weak peak arose at 821 cm^−1^, which might be associated with the C–H stretching of aromatic rings.

In general, alcohols, polyphenols, and amines functional groups found in the *Cynoglossum creticum* leaf extract contribute to the reduction and stabilization processes. The hydroxyl groups in alcohols and polyphenols have a high propensity to chelate silver ions, while the carbonyl group can surround and link to metals more effectively than proteins and amino acids[78]. Proteins bind to the produced silver particles via free amino groups, providing a surface coating that efficiently protects the AgNPs from aggregation in solution and ensures long-term stability[79].

#### XRD Analysis

**Fig. 4:**
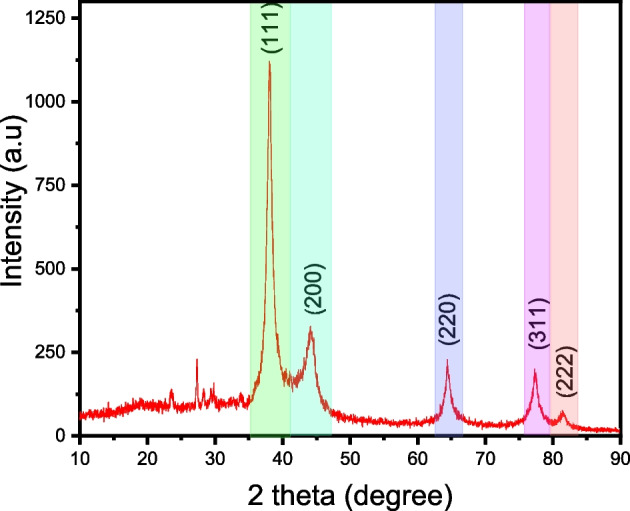
XRD pattern of the green-synthesized Ccl-AgNPs

The X-ray diffraction pattern displayed in Fig. 4 shows silver crystal existence in biosynthesized AgNPs. Five major diffraction peaks at (2θ) = 38.15, 44.10, 64.39, 77.30, and 81.57 may be linked to the (111), (200), (220), (311), and (222) planes, respectively, referring to the face-centered cubic structure of silver (fcc) (ICSD file no. 98–005–3761). The peak corresponding to the (111) plane is the most intense of the related planes. This implies that the predominant growth of AgNPs was directed along the (111) direction[63]. The crystallite size of the developed silver nanoparticles was estimated employing the Debye–Scherrer formula (Eq. 1).

1 $$D=K\lambda /\beta \mathrm{cos}\theta$$

where *λ* is the X-ray source wavelength applied in XRD, *K* is the Scherrer constant, *β* is the full width at half maximum (FWHM) of the peak of the (111) plane, *θ* is the Bragg angle, and *D* is the crystallite size of AgNPs, which was apparent to be 24.3 nm.

#### Morphological Examination (SEM–EDX)

**Fig. 5:**
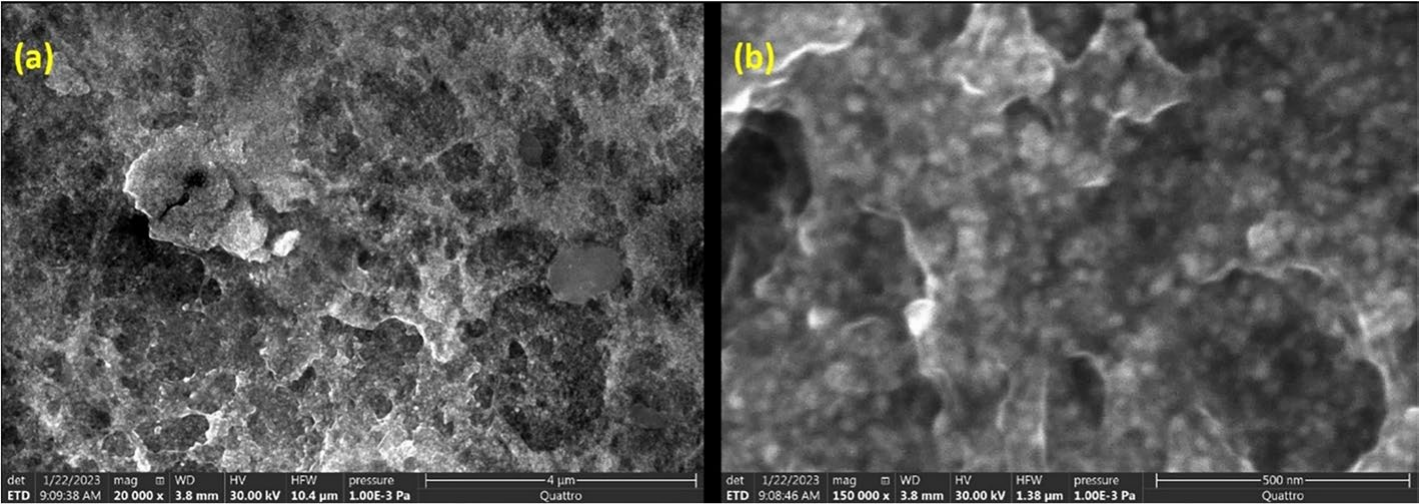
**a** and **b** SEM micrograph of Ccl-AgNPs at different magnifications

Figure 5a and b display the micrographs of the biosynthesized Ccl-AgNPs at different magnifications. The SEM image taken at low magnification (4 µm) demonstrates an overall structure consisting of a clustering of particles that can be assigned to interaction between the concentrated nanoparticles and organic substances involved in biosynthesis[80]. A high-magnification SEM image (Fig. 5b) reveals the presence of white spots, confirming that the particles were somewhat spherical in shape, which is accordant with the UV–visible spectroscopy results above. Additionally, there are certain agglomerations possibly caused by the evaporation of the solvent during the colloidal synthesis or Ccl-AgNPs’ powdered process by centrifugation and drying[81].

ImageJ software served for assessing the dimensions of the nanoparticles from the SEM results[82]. Figure S5(a) presents the particle size histograms for Ccl-AgNPs; the particle size varied from 15 to 60 nm with an average diameter of about 32 nm. The estimated average size is close to the XRD analysis outcome. In fact, the Scherrer formula applied to the XRD peak of the (111) plane yields information about the crystallite size[83]. SEM images reveal the size of the particles, and a single particle can be formed from one or more crystallites. This could explain the slight difference between the SEM and XRD analyses outcomes.

AgNPs elemental composition and crystalline nature were verified by EDS analysis. It is common knowledge that Ag spherical nanoparticles display optical absorption peaks in the range 2.7–3.4 keV owing to surface plasmon resonances[84]. Figure S5(b) showed the existence of silver peaks at around 3 keV, affirming the successful synthesis of crystalline silver NPs. In addition to silver, strong signals of carbon and oxygen from organic sources also appeared in the EDS spectrum, corresponding to the secondary metabolites of Ccl-extract. It was found that the weight percentages of silver, carbon, and oxygen are 24.68, 30.48, and 44.85%, respectively (Table S2), which confirms the formation of organo-metallic nanoparticles consisting of silver covered with a layer of phytochemicals[85].

#### Thermogravimetric Analysis (TGA)

**Fig. 6:**
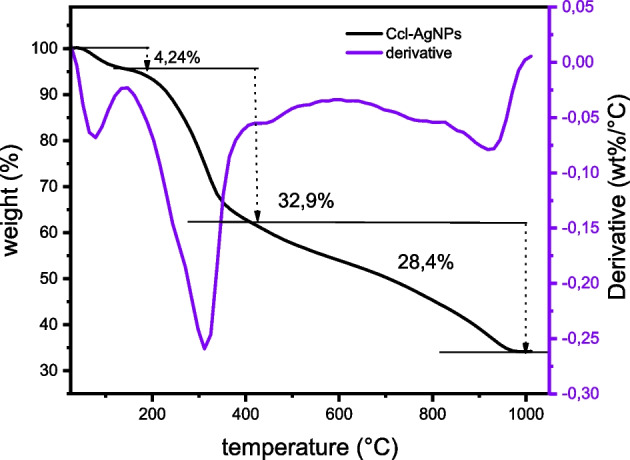
TGA-DTA thermogram of Ccl-AgNPs

Thermogravimetric analysis (TGA) was done to comprehend the behavior and stability of biologically produced nanomaterials under different temperature conditions, as well as to prove the presence of organic material on their surface as a capping agent. The thermogram (Fig. 6) revealed multiple decompositions of Ccl-AgNPs samples, with three weight loss patterns caused by the evolution of moisture, the rupturing of chemical bonds, and secondary metabolite molecule breakdown[86]. A slight loss in weight of 4.24% was recorded at a temperature lower than 133 °C due to moisture and volatile substances on the Ccl-AgNPs surface. The second mass loss was dominant at 32.9% between 133 and 400 °C, which may be due to the thermal degradation of plant organic biomolecules over the AgNPs surface, followed by a weight loss of 28.4% above 400 °C associated with resistant aromatic and oxygen compounds[87]. TGA data not only offer insights on the thermal stability of nanoparticles but also allow for the evaluation of the yield of biosyntheses of AgNPs[88]. At the final temperature of 1000 °C, 34.46% of the weight remained as pure silver metal.

### Antioxidant Activities of Ccl-AgNPs

Secondary metabolites, including flavonoids and phenolic compounds, participate in the bioreduction of silver ions, as their concentration determines not only the reaction kinetics but also the synthesized nanoparticle shape and size, as they also stabilize the nanoparticles in the colloidal system[15]. The total content of each phenol and flavonoid in the Ccl-AgNPs was quantified as gallic acid equivalent and quercetin equivalent, respectively, and the results showed that Ccl-AgNPs contained a high level of polyphenols and flavonoids (TPC, 246.15 ± 6.73 µg AcG/mg Ccl-AgNPs; TPF, 143.61 ± 3.5 µg QEs/mg Ccl-AgNPs). The high amount of total phenolic and flavonoid in the structure of the as-synthesized Ccl-AgNPs supports the assembly of Ag zero-valent metal into smaller AgNP[60].

To obtain more trustworthy results, four distinct approaches were employed to determine the antioxidant properties of Ccl-AgNPs: DPPH, ABTS, reducing power, and phenanthroline assay. This is owing to the high content of complex phytochemicals in the Ccl-AgNPs samples, which can perform various antioxidant mechanisms. Table 1 displays the antioxidant potential in terms of IC_50_ and A_0.5_ values.

**Table 1:** Antioxidant activities of the biosynthesized Ccl-AgNPs

Samples	DPPH assay	ABTS assay	Reducing power assay	Phenanthroline assay
	IC_50_ (µg/mL)	IC_50_ (µg/mL)	A_0.50_ (µg/mL)	A_0.50_(µg/mL)
Ccl-AgNPS	20.62 ± 0.13	39.25 ± 3.51	123.66 ± 2.48	26.17 ± 6.22
BHA	6.14 ± 0.41	1.29 ± 0.30	8.41 ± 0.67	0.93 ± 0.07
BHT	12.99 ± 0.41	1.81 ± 0.10	NT	2.24 ± 0.17
*α*-Tocopherol	13.02 ± 5.17	NT	34.93 ± 2.38	NT
Ascorbic acid	14.47 ± 0.61	NT	10.41 ± 1.40	5.86 ± 0.14
Catechin	NT	NT	28.98 ± 1.17	4.84 ± 0.04

IC_50_ and A_0.50_ values are stated as means ± SD of three measurements

NT: Not Tested

As evident by the DPPH scavenging results, Ccl-AgNPs present noticeable antioxidant activity with IC_50_ values of 20.62 ± 0.13 µg/mL. Nevertheless, this activity is comparatively less than that of the standards BHA (6.14 ± 0.41), BHT (12.99 ± 0.41), *α*-tocopherol (13.02 ± 5.17), and ascorbic acid (14.47 ± 0.61). Also, Ccl-AgNPs likewise showed the capacity to decolorize the blue-green chromophore ABTS^+^ with an IC_50_ of 39.25 ± 3.51 µg/mL, which is less than that of BHT and BHA (IC_50_, 1.29 ± 0.30 and 1.81 ± 0.10 µg/mL, respectively). The Ccl-extract-coated AgNPs displayed moderate ABTS cation scavenging activity when compared to the antioxidant standards used.

Reducing power and phenanthroline tests were performed to assess as-prepared silver nanoparticles’ capacity to reduce ferric iron by donating electrons. Monitoring of these reduction reactions relies on a colorimetric indicator, as reducer compounds convert the yellow-colored Fe^+3^ into the blue-green Fe^+2^ in a reducing power assay. As for the 1,10-phenanthroline technique, the orange [Fe(Phen)3]^3+^ is reduced to a more stable orange-red complex [Fe(Phen)3]^2+90^. The ferric-reducing ability of Ccl-AgNPs was found to be (A_0.5_ = 123.66 ± 2.48 µg/mL), weaker than that recorded for positive controls (see Table 1). Ccl-silver nanoparticles demonstrated good activity in the phenanthroline assay, with an *A*_0.50_ amount of 26.17 ± 3.08 µg/mL. In contrast, the activity of the standards, ascorbic acid, catechin, BHT, and BHA, was spotted to be stronger in reducing ferric iron in the presence of 1,10-phenanthroline, with *A*_0.50_ values of 5.86 ± 0.14, 4.84 ± 0.04, 2.24 ± 0.17, and 0.93 ± 0.07 µg/mL, respectively.

Biosynthesized silver nanoparticles using *Cynoglossum creticum* leaf extract proved to be an active antioxidant, probably due to the redox properties of the phenolic components over their surface. In actuality, there was a simultaneous activity between these natural redactors capable of readily donating electrons and hydrogen atoms and the AgNPs as a catalyst, allowing the colloidal Ccl-AgNPs system to scavenge free radicals and chelate metals[90, 91]. Dauthal and Mukhopadhyay[92] propose that the enhanced antioxidant properties of green-synthesized nanoparticles are due to the phenomenon of surface interaction, in which the existence of antioxidant substances absorbed on the active surface of nanoparticles and their large surface area to volume ratio creates an ability to interact and inhibit free radicals.

### Antibacterial Activities of Ccl-AgNPs

For years, research has confirmed the excellent antimicrobial abilities of AgNPs against drug-resistant pathogenic bacteria[11, 66]. Biosynthesis could be a viable alternative process that contributes to reducing the potential effects of AgNPs on safety and ecology. In this current investigation, the antibacterial activity of *Cynoglossum creticum* leaves’ extract-coated silver nanoparticles against four ATCC microorganism strains was examined, employing the microdilution approach. Table 2 displays the results of MIC values, where it shows that the highest inhibitory impact of Ccl-AgNPs was against Gram-negative *P. aeruginosa* with a MIC value of 31.25 µg/mL. For the other test bacteria strains, *E. coli*, *E. faecalis*, and *S. aureus*, 62.5 µg/mL of Ccl-AgNPs was the minimum inhibitory concentration.

**Table 2:** Minimal inhibitory concentration (MIC) of Ccl-AgNPs against bacterial strains

Ccl-AgNPs concentration (µg/mL)	Bacterial growth			
	*E. faecalis* ATCC 29212	*S. aureus* ATCC 29213	*E. coli* ATCC 25922	*P. aeruginosa* ATCC 27853
15.625	+	+	+	+
31.25	+	+	+	−
62.5	−	−	−	−
125	−	−	−	−
250	−	−	−	−
500	−	−	−	−
1000	−	−	−	−
MIN	62.5	62.5	62.5	31.25

Positive signs (+) indicate bacterial growth, and negative signs (−) no bacterial growth

These findings confirm the remarkable potency of Ccl-AgNPs in inhibiting the growth of both Gram-negative and Gram-positive bacterial strains. However, the antibacterial activity of Ccl-AgNPs was more effective against Gram-negative bacteria rather than Gram-positive bacteria, as *P. aeruginosa* was the most vulnerable to Ccl-AgNPs among the bacterial strains utilized in the investigation. These results correlate with previous reports that plant extract-mediated synthesized AgNPs possess better antibacterial activity toward Gram-negative bacteria than Gram-positive bacteria[77, 78, 81].

Although numerous studies have attempted to discover the exact mechanism of the antibacterial action of AgNPs, it is still not entirely comprehended[93]. In general, there is an electrostatic attraction between the silver ions released from AgNPs and the phosphorus and thiol groups present in the bacterial cell membrane and wall, which raise the permeability, causing membrane structures to alter and rupture[13, 87]. Unlike Gram-negative bacteria, Gram-positive bacteria have solid layers of peptidoglycan that attract fewer silver ions, resulting in a limited antibacterial effect[11, 88]. The large surface area of AgNPs obtained from their nano-size and spherical/semi-spherical shape contributes to improved contact with microorganisms and penetration into them[93]. Ag^+^ ions absorbed inside the cell stimulate the creation of reactive oxygen species (ROS), harmful free radical species, and facilitate the release of adenosine triphosphate (ATP), causing oxidative stress and respiratory chain disorders and preventing deoxyribonucleic acid (DNA) replication and cell proliferation[13, 86, 87].

### Utiliztion of Ccl-AgNPs as a Colorimetric Sensing Probe

**Fig. 7:**
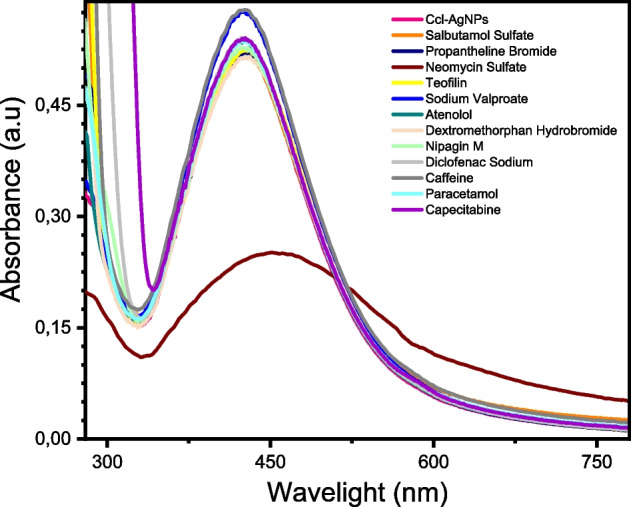
UV–vis spectra of Ccl-AgNPs after adding drug solutions

The applicability of *Cynoglossum creticum*-assisted synthesized AgNPs as a potential sensing probe toward different drugs was evaluated. Separately, the Ccl-AgNPs solution was treated with a fixed concentration of various drugs, and the modification in UV–vis spectra of Ccl-AgNPs prior to and after was measured. As Fig. 7 illustrates, with the exception of neomycin sulfate, there was no noticeable alteration in the SPR range of Ccl-AgNPs after other drugs' addition. Treatment of Ccl-AgNPs with NEO resulted in a considerable reduction in absorption intensity with a red shift in the maximum absorption wavelength from 424 to 451 nm. This came with visible color changes, as the brown color of the colloidal Ccl-AgNPs solutions darkened within a few minutes after the addition of NEO solution, and a brown precipitate subsequently formed due to the agglomeration of the Ccl-AgNPs (see Fig. S6). Based on preliminary screening results, Ccl-extract stabilized AgNPs perform as a selective plasmonic probe for neomycin detection.

FTIR and SEM outcomes were performed to further understand Ccl-AgNPs’ agglomeration mechanism and determine the nature of the interactions between the antibiotic neomycin and Ccl-AgNPs. SEM images of the morphology of the NEO-Ccl-AgNPs (Fig. S7) demonstrate that the presence of neomycin led to the nanoparticles getting closer together and agglomerating. This explains the decrease in surface plasmon resonance absorption and its shift to higher wavelengths in the UV–vis spectrum, confirming colorimetric sensing based on the silver nanoparticles’ agglomeration concept[94, 95].

FT-IR spectra of NEO, Ccl-AgNPs, and NEO-Ccl-AgNPs were recorded and presented in Fig. S8. The FTIR spectrum of neomycin sulfate exhibits a distinctive broad diffuse band ranging between 3600 and 2400 cm^−1^ owing to the vibrations of numerous organic groups, including the stretching vibrations of the hydroxyl (− OH) and amino (N–H) groups between 3600 and 3100 cm^−1^ and the stretching vibrations of the alkyl (C–H) between 3000 and 2800 cm^−1^^97^.[96] The spectrum also shows other characteristic peaks at 1618 and 1521 cm^−1^ that are linked to N–H bending vibrations of cycloaliphatic amine. A strong absorption peak at 1018 cm^−1^ corresponds to C–N stretching vibrations, and a peak at 606 cm^−1^ that is mostly due to the bending vibration of sulfur dioxide (SO_2_) [97, 98].Interestingly, the spectrum of the NEO-Ccl-AgNPs complex showed both features of the spectra of NEO and Ccl-AgNPs, with some minor changes. The characteristic aromatic (C = C) band from Ccl-extract in the FTIR spectra of Ccl-AgNPs disappeared in the NEO-Ccl-AgNPs spectrum, while N–H bending peaks of NEO at 1618 and 1521 cm^−1^ were shifted to 1595 and 1510 cm^−1^, highlighting the contribution of benzene and nitrogen groups in the reaction[99]. There was an obvious change in the range from 2800 to 3000 cm^−1^ in the NEO-Ccl-AgNPs spectrum compared to Ccl-AgNPs due to alteration in the stretching attitude of CH_3_ and CH_2_. The O–H band was broadened after treatment of Ccl-AgNPs with NEO, which may refer to the development of hydrogen bonding between the hydrogen of the hydroxyl and amino groups of neomycin and the OH groups of Ccl-AgNPs[98, 100]. Through comparing the FTIR spectra, the agglomeration of NEO-Ccl-AgNPs is likely to have resulted from interactions between the polyphenols and proteins on the Ccl-AgNPs surface and neomycin molecules.

Ccl-AgNPs sensitivity towards NEO was assessed by performing quantitative measurements based on titrating different concentrations (0.01–200 µM) of NEO with Ccl-AgNPs solutions and recording the changes in SPR band intensity. The calibration curve of absorbance at 424 nm as a function of NEO concentration (Fig. S10) shows an excellent linear relationship with a regression constant *R*^2^ of 0.998 in the range of 0.5–50 µM. The limit of detection (LOD) for NEO was reckoned from the calibration graph employing the formula LOD = 3.3 × (SD of intercept/slope), and the LOD was found to be 1.81 µM[101]. Moreover, a Job plot was created to estimate the binding stoichiometry of Ccl-AgNPs and NEO (Fig. S11). The maximum binding was seen at a molar ratio of 0.5, indicating an equimolar binding ratio (1:1) between Ccl-AgNPs and NEO.

The most important aspect for the actual function of every sensing probe is its selectivity toward the analyte in the presence of coexistent compounds and additional interferences[102, 103]. In this regard, an interference study done to assess Ccl-AgNPs selectivity towards NEO using different organic and inorganic interferences commonly existing in environmental and biological samples, including the main ions in drinking water and blood plasma (Na^+^, Ca^2+^, K^+^, NH^4+^, Mg^2+^, Cl^−^, CO_3_^2−^, and SO_4_^2−^) [69, 104], blood constituents such as fructose, lactose, maltose, ascorbic acid, and urea, and anticoagulants like ethylenediaminetetraacetic acid (EDTA) and trisodium citrate[105, 106]. Each interferent (1 mM) and NEO solution (1 mM) was added to the Ccl-AgNPs in equal ratios of 1:1:1 (v/v/v). Results in Fig. S12 revealed that the presence of potential interferences did not contribute any noticeable modifications in the absorption intensity of the neomycin-Ccl-AgNPs complex, indicating that only NEO induces Ccl-AgNPs agglomeration. These results demonstrate that the colorimetric Ccl-AgNPs sensor possesses high selectivity for neomycin detection in the occurrence of natural interferences. Ccl-AgNPs-based sensing system performance in the detection and quantification of NEO was verified in real matrices like tap water, rabbit blood plasma, and veterinary drug formulations. Plasma samples were diluted in distilled water before use in order to reduce the matrix effect, and water samples were used directly without further treatment. Biological and environmental samples were spiked with various doses of NEO (10, 30, and 50 µM) and then analyzed according to the Ccl-AgNPs colorimetric method. Results of UV–vis spectra (Figs. S13 and S14) showed a reduction in absorption intensity and a red shift in the SPR band of Ccl-AgNPs mixed with spiked samples, confirming the selective recognition of NEO. Quantification of NEO in biological and environmental samples and veterinary preparations was also performed by recovery measurements (Table S3). The recoveries of NEO (%) range between 93.2 and 104.0 for plasma samples, 92.62–97.3 for tap water samples, and 94.55–105.76% for veterinary drug formulations. The recovery data demonstrate the reliability of the colorimetric sensing method and its practicability in determining NEO in various systems.

## Conclusion

In this work, the potential of *Cynoglossum creticum* leaves as a reducing and capping agent in a safer and eco-friendly biosynthesis of silver nanoparticles (Ccl-AgNPs) was explored. The synthesis process was improved using a one-factor-at-a-time strategy (ratio of reagents, incubation time, and temperature) to achieve the highest yield of Ccl-AgNPs with good stability and dispersibility. The optical, crystal structure, morphological, compositional, and thermal properties of the as-synthesized Ccl-AgNPs were analyzed through multiple approaches, involving UV–vis, FTIR, XRD, SEM–EDS, and TGA. Moreover, the green-synthesized Ccl-AgNPs exhibited potential biological activities, as antioxidant content assays revealed the promising ability of Ccl-AgNPs to scavenge free radicals and reduce ferric iron. While an antibacterial activity test revealed that Ccl-AgNPs are effective in inhibiting pathogenic strains, especially Gram-negative bacteria. The possibility of applying Ccl-AgNPs in SPR-based colorimetric sensing for quantitative detection of neomycin sulfate in biological, ecological, and veterinary pharmaceutical matrices was also verified. The biogenic Ccl-AgNPs detection system displayed high and rapid sensitivity and selectivity for neomycin sulfate in the presence of organic and inorganic interferers. The synthesis potential of *Cynoglossum creticum* extract (a livestock-hazardous weed), combined with the biological and sensing features of Ccl-AgNPs, enables the development of a simple and effective green nanotechnology for potential health and environmental applications, realizing the concept “waste to wealth.”

## Supplementary Information

Below is the link to the electronic supplementary material.

ESM1 (DOCX 2.02 MB)

## Data Availability

The data will be available upon request to the authors.
