# Supplementary material for: Green Synthesis of Silver Nanoparticles Using Cynoglossum creticum Leaf Extract: Eco-Friendly Approach for Antibacterial, Antioxidant, and Sensing Applications
Source: Appl Biochem Biotechnol. 2025 Oct 23;197(12):7981–8007. doi: 10.1007/s12010-025-05390-2 (PMC12718234; doi:10.1007/s12010-025-05390-2)
Supplement: Supplementary file 1 — (DOCX 2.02 MB) [file 12010_2025_5390_MOESM1_ESM.docx]

**Green Synthesis of Silver Nanoparticles Using *Cynoglossum creticum* Leaf Extract: Eco-Friendly Approach for Antibacterial, Antioxidant, and Sensing Applications**

**Seif El Islam Boudagha^1^, Chafia Sobhi^2^, Hamdi Bendif^3^*, Emel Öykü Çetin Uyanikgil^4^, Amdjed Abdennouri^5^, Mustafa Ökeer^6^, Chawki Bensouici^7^, Moussa Boudiaf^8^, Ahmed Zouaoui^9^,** **Hassan A Rudayni^3^, Fehmi Boufahja^3^, Stefanıa Garzoli ^10^****

**TABLE S 1.** Preliminary Phytochemical screening of *Cynoglossum creticum* leaves extract.

| **Phyto-constituents** | **Chemical tests** | **Result** |
| --- | --- | --- |
| tannins | Ferric chloride test | + |
| alkaloid | Wagner’s reagent | + |
| saponins | Foam test | + |
| flavonoids | Sodium Hydroxide test | + |
| quinones | Sulfuric acid test | + |
| anthraquinon | Borntragor’s Test | - |
| sterols /terpenes | Liebermann-Buchard test | + |


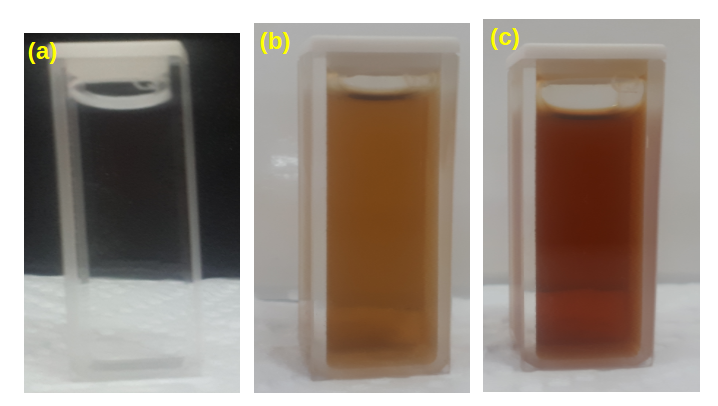


**FIGURE S1.** (a) Silver nitrate, (b) Ccl-extract, and (c) Ccl-AgNPs.

**

**FIGURE S2**.UV-visible spectrum of Ccl-AgNPs synthesized under optimized conditions.

**FIGURE S3.** UV-vis spectra of Ccl-AgNPs in the presence of various concentration of NaCl.

**FIGURE S4.** UV-visible spectra of Ccl-AgNPs at different pH*.*


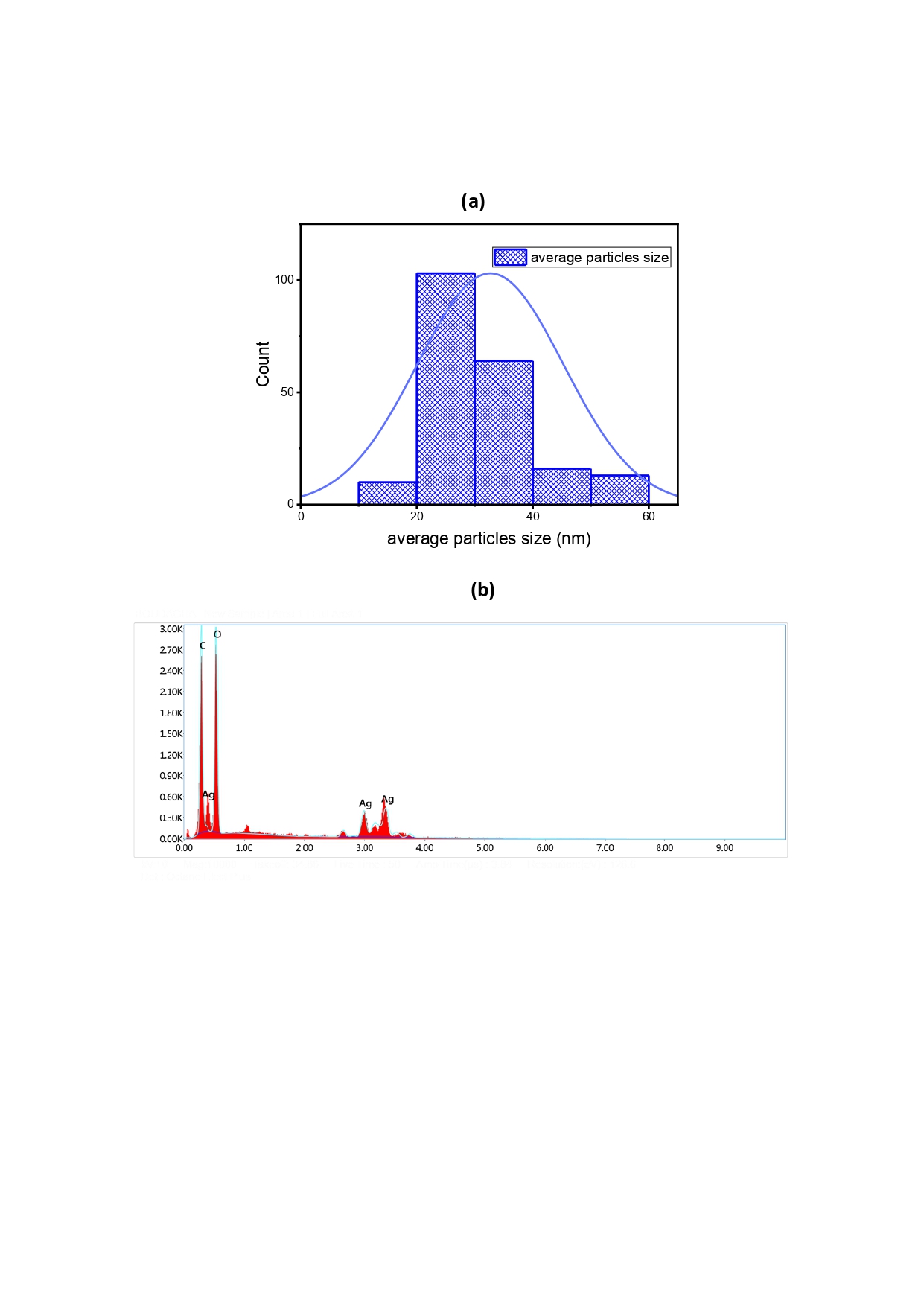


**FIGURE S5.** (a) particle size distribution histogram, and (b) EDS spectrum of Ccl-AgNPs.

**TABLE S2.** Elemental composition of green synthesized Ccl-AgNPs

| **Element** | **Weight %** | **Atomic %** | **Error %** | **Net Int.** | **R** | **A** | **F** |
| --- | --- | --- | --- | --- | --- | --- | --- |
| C K | 30.48 | 45.56 | 8.01 | 269.87 | 0.8871 | 0.4730 | 1.0000 |
| O K | 44.85 | 50.33 | 9.65 | 312.83 | 0.9012 | 0.3131 | 1.0000 |
| Ag L | 24.68 | 4.11 | 4.85 | 74.89 | 0.9524 | 0.9663 | 1.0040 |


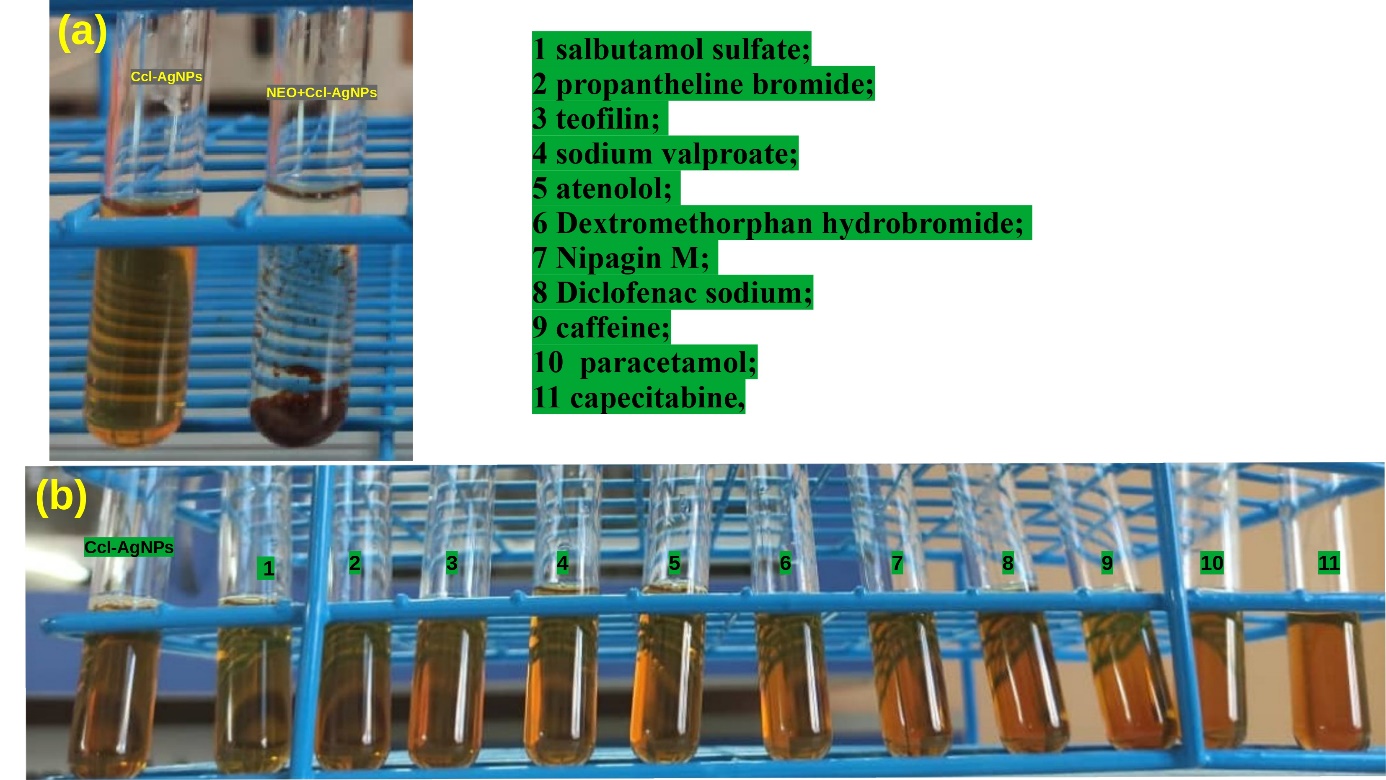


**FIGURE S6.** Change of the color of the colloidal Ccl-AgNPs ; (a) in the presence of neomycin sulfate, and (b) in the presence of the rest of the tested drugs.


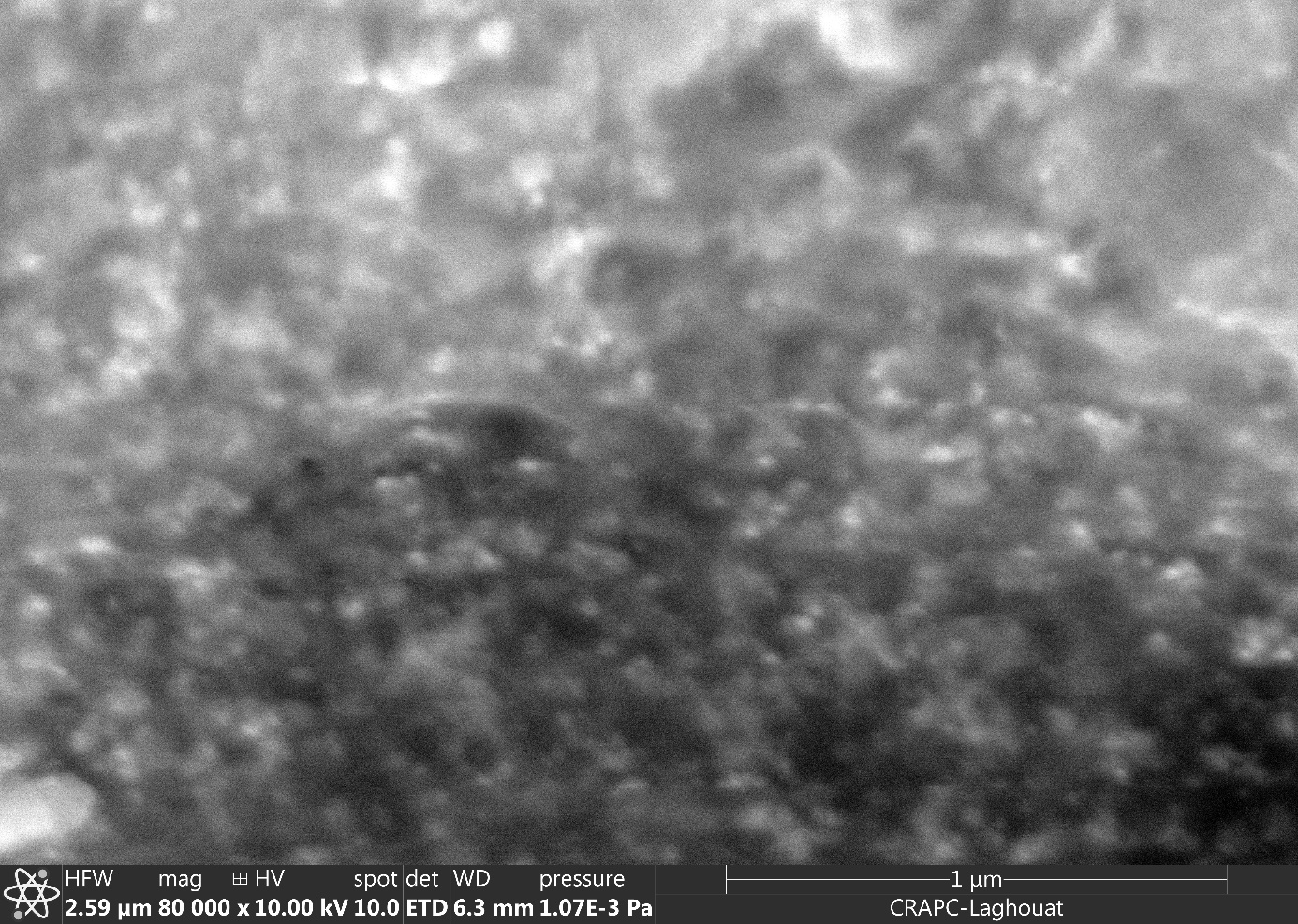


**FIGURE S7.** SEM images of NEO-Ccl-AgNPs.

**FIGURE S8.** FTIR spectrum of neomycin sulfate (NEO), Ccl-AgNPs , and NEO-Ccl-AgNPs.

**FIGURE S9.** UV–vis spectra of Ccl-AgNPs treated with different concentrations of NEO, and (b) the diagram of absorbance intensity A_424_ as a function of NEO concentration.

**FIGURE S10.** diagram of absorbance intensity A_424_ as a function of NEO concentration.

**FIGURE S11.** Job plot for binding ratio of Ccl-AgNPs and NEO.

**FIGURE S12.** Effect of the presence of interfering substances on the selectivity of Ccl-AgNPs toward NEO.

**FIGURE S 13.** NEO recognition in a real environmental sample (tap water).

**FIGURE S 14.** Detecting of NEO in a biological sample (animal blood plasma).

**TABLE S3.** Quantitative analysis of NEO in environmental and biological and veterinary drug formulation samples.

| **Sample** | **Concentration of the added NEO (µM)** | **Calculated Concentration (µM)** | **Recovery%** |
| --- | --- | --- | --- |
| Tap Water | 10 | 9.73 | 97.3 |
|  | 30 | 27.78 | 92.62 |
|  | 50 | 48.17 | 96.34 |
| Plasma | 10 | 9.326 | 93.27 |
|  | 30 | 28.20 | 94.01 |
|  | 50 | 52.01 | 104.03 |
| veterinary drug formulation | 10 | 10.57 | 105.76 |
|  | 30 | 28.36 | 94.55 |
|  | 50 | 50.86 | 101.73 |
